# Bi-Level Optimization for De-Icing Position Allocation and Unmanned De-Icing Vehicle Fleet Routing Problem

**DOI:** 10.3390/biomimetics9010026

**Published:** 2024-01-03

**Authors:** Jian Liu, Qi Huang, Yuhang Han, Shiyun Chen, Nan Pan, Renxin Xiao

**Affiliations:** 1 Faculty of Civil Aviation and Aeronautics, Kunming University of Science and Technology, Kunming 650500, China; 20222245022@stu.kust.edu.cn (J.L.); 202114506239@stu.kust.edu.cn (Q.H.); hanyuhang@stu.kust.edu.cn (Y.H.); 20232245034@stu.kust.edu.cn (S.C.); 2 Faculty of Transportation Engineering, Kunming University of Science and Technology, Kunming 650500, China; xiaorenxin@kust.edu.cn

**Keywords:** de-icing positions, unmanned de-icing vehicle, genetic algorithm, bi-level optimization

## Abstract

Aircraft icing due to severe cold and local factors increases the risk of flight delays and safety issues. Therefore, this study focuses on optimizing de-icing allocation and adapting to dynamic flight schedules at medium to large airports. Moreover, it aims to establish a centralized de-icing methodology employing unmanned de-icing vehicles to achieve the dual objectives of minimizing flight delay times and enhancing airport de-icing efficiency. To achieve these goals, a mixed-integer bi-level programming model is formulated, where the upper-level planning guides the allocation of de-icing positions and the lower-level planning addresses the collaborative scheduling of the multiple unmanned de-icing vehicles. In addition, a two-stage algorithm is introduced, encompassing a Mixed Variable Neighborhood Search Genetic Algorithm (MVNS-GA) as well as a Multi-Strategy Enhanced Heuristic Greedy Algorithm (MSEH-GA). Both algorithms are rigorously assessed through horizontal comparisons. This demonstrates the effectiveness and competitiveness of these algorithms. Finally, a model simulation is conducted at a major northwestern hub airport in China, providing empirical evidence of the proposed approach’s efficiency. The results show that research offers a practical solution for optimizing the use of multiple unmanned de-icing vehicles in aircraft de-icing tasks at medium to large airports. Therefore, delays are mitigated, and de-icing operations are improved.

## 1. Introduction

In cold weather conditions, de-icing aircraft is a crucial procedure. This process ensures the removal of ice and snow accumulated on the aircraft’s surface, ensuring a safer flight. With the recovery of the civil aviation industry, the significant increase in airport flights has accentuated the deficiency of de-icing resources, particularly in extreme weather conditions [1]. In cold weather conditions, aircraft awaiting takeoff may be susceptible to icing. This phenomenon can occur for two reasons: during the preceding flight missions, the aircraft’s surfaces may accumulate ice due to the impact of low-temperature water droplets, a condition known as in-flight icing; alternatively, the aircraft’s surfaces may ice up when exposed to freezing precipitation at the airport. The accretion of ice on an aircraft can alter its aerodynamic profile, thereby impacting its aerodynamic performance. This poses a significant threat to flight safety and, in extreme cases, can lead to fatal accidents [2]. Moreover, statistical data reveal that over 15% of aviation accidents are attributed to aircraft icing [3]. It is evident from the above that icing on aircraft significantly impacts flight safety. De-icing the aircraft before takeoff is a crucial step to ensure safety. One challenge that researchers are focusing on is methodology to improve airport de-icing efficiency when resources are limited. Specifically, the requirements of de-icing resource management involve allocating flights that require de-icing to limited positions while aligning with airport operations. Furthermore, unmanned de-icing vehicles are gradually replacing traditional de-icing vehicles as carriers of de-icing fluid. Therefore, the allocation of de-icing positions and the scheduling of unmanned de-icing vehicles are primary factors in optimizing airport de-icing resources.

Current airport de-icing operations can be primarily classified into three categories: slow-movement de-icing, gate de-icing, and centralized de-icing. The first category establishes positions close to the runway or takeoff section. Once the aircraft enters these positions, it is de-iced by vehicles while the engines remain in an idle state. This method requires that the vehicles operate within the jet wash area of the plane, therefore increasing the risk factor. Due to this heightened danger, few airports apply this mode of de-icing [4]. The second category (i.e., gate de-icing), also known as dispersed de-icing, consists of keeping the aircraft stationary in its position while the vehicles approach it for de-icing. Several challenges are associated with this approach, such as the dispersed locations of vehicle operations, the longer distances from fluid refilling points, and environmental concerns due to the impossibility of efficiently recovering the used de-icing fluid [5]. Finally, the third category (i.e., centralized de-icing) consists of de-icing the aircraft collectively in a specific area of the runway. Such an approach is typically employed at medium to large airports. Without compromising airport interests, centralized de-icing can significantly conserve vehicle resources. In particular, when resources are scarce, it effectively enhances the airport’s de-icing processes. This method enables staff to arrange de-icing tasks based on the actual number of flights awaiting de-icing and the available resources and to convey resource dispatch strategies accordingly. For instance, Dallas—Fort Worth International Airport (DFW) has implemented a centralized de-icing strategy, reducing the average waiting time for winter de-icing per flight from 9 to 4 min. Furthermore, airports in China, such as Capital International Airport, Tianjin Binhai International Airport, and Beijing Daxing International Airport, have also implemented centralized de-icing strategies. In a study by Chenbin [6], simulations were realized using actual flights as well as de-icing resource data from Beijing Capital International Airport. The results showed that for a target time period involving 30 flights, the improved centralized scheduling strategy proposed in the work reduced the total flight delay time by 732 min compared to the first-come-first-served strategy where one de-icing position was open at a time.

Moreover, centralized de-icing scheduling strategies can be further divided into step-by-step scheduling and integrated scheduling. The former first allocates de-icing positions and then dispatches vehicles based on the results of these allocations. Although this strategy maximizes the benefits for the control center, it tends to overlook the interests of ground service companies. As for the latter (i.e., integrated scheduling), it examines the coordinated dispatching of both de-icing positions and vehicles [6]. While safeguarding the interests of the control center, it concurrently considers the advantages for ground service companies. Moreover, it has the potential of optimizing the use of de-icing resources when faced with scarcity. However, the implementation of this approach is challenging due to the necessity for collaboration and information sharing among participants. The essence of the integrated scheduling strategy is centered on establishing a link between the allocation of de-icing positions and the deployment of unmanned de-icing vehicles. Therefore, recognizing the mutual influences on both aspects is vital to maximize the benefits for ground service companies while ensuring minimal flight delay.

The centralized de-icing resource scheduling strategy primarily consists of two steps: the allocation of de-icing positions and dispatching of de-icing vehicles. The allocation of de-icing positions can be considered as a task assignment problem that is foundational in combinatorial optimization, aiming to assign one or multiple tasks to various agents while minimizing assignment costs [7,8]. In the aviation field, there is limited research focusing on the de-icing position assignment problem; moreover, most studies are centered on flight allocation issues and boarding gate assignments. However, when assigning flights, it is essential to consider several factors, such as delay costs, airport capacity, and position exchanges between airlines [9] as well as passenger costs [10]. For instance, Kim et al. [11] proposed an approach to solve aircraft landing problems by minimizing the total penalty cost due to deviations from target landing times, while working within constraints that are presented within specified time windows. Concerning gate assignment issues, Li et al. [12] introduced a column generation method to address the airport flight arrival assignment problem, aiming to optimally allocate each scheduled flight to a compatible gate in order to minimize ground delays upon arrival. In addition, Mei et al. [13] considered several factors, such as transfer speed, transfer demands, flight size, parking position dimensions, and terminal configurations, and optimized the sequence of flights and parking position allocations to minimize the total costs. In addition, She et al. [14] appropriately assigned various flight activities, including arrivals, parking, and departures, to different boarding gates during operation. Furthermore, Silva et al. [15] combined gate assignment problems with passenger behavior modeling as they considered passenger experience and economic viability by establishing a Mixed Integer Linear Programming (MILP) model to maximize non-aeronautical revenue for terminals. Moreover, Sßeker et al. [16] dealt with the gate assignment issue under uncertain flight arrival and departure times. They built a stochastic planning model that incorporated robust measurements based on the number of conflicting flights, free time, and buffer time.

To sum up, research on the aforementioned task assignment issues in the aviation domain is primarily concentrated on flight takeoffs and landings, boarding gate allocations, and parking position assignments. However, the principles of parking position allocation differ from those of de-icing position allocation. In the first case, one also needs to consider the constraints posed by de-icing resources. Therefore, the allocation models for the mentioned scenarios cannot be applied directly to de-icing position assignments as this requires the exploration of new allocation schemes.

Therefore, the scheduling of unmanned de-icing vehicles is typically considered a Vehicle Routing Problem (VRP). Research related to this topic is relatively infrequent; however, insights can be drawn from the scheduling of other specialized vehicles within the airport. For instance, for baggage transfer vehicles within the airport, Zhang et al. [17] considered real-world conditions, including split demands, multiple trips, and concurrent pickups and deliveries. They employed topological sorting to address these intricate dependencies and define the start time for each service in order to efficiently schedule vehicles. Moreover, for airport shuttle buses aiming to reduce the weighted sum of flight delays, Lv et al. [18] proposed a Variable Neighborhood Search (VNS) algorithm to optimize the model. To decrease the operational energy consumption of these shuttles, Sigler et al. [19] introduced a discrete event simulator that evaluates the optimal shuttle routes in stochastic settings and identifies the trade-off between passenger waiting times and shuttle energy consumption. Moreover, VRP research has yielded significant findings in various domains [20], as these solutions refer to the scheduling of specialized vehicles within airports. However, vehicular scheduling in this context is distinct from other scheduling problems; it must be based on the results of flight scheduling, as changes in flight schedules directly influence vehicle scheduling plans. The same procedure is valid for unmanned de-icing vehicle scheduling: alterations in flight assignments directly affect the scheduling plan for unmanned de-icing vehicles. Consequently, a collaborative approach between de-icing position allocation and unmanned de-icing vehicle scheduling is needed.

The collaborative problem solving of de-icing position allocation and unmanned de-icing vehicle scheduling can draw inspiration from the methods employed in coordinating airport surface resources. For instance, Zhang et al. [21] developed a multi-agent framework for coordinating general aviation resources, creating a capacity resource matching model using the auction mechanism to enhance parallel task processing capabilities. They devised resource scheduling strategies based on the matching results and validated the feasibility of the proposed method using real operational data. Moreover, Chen et al. [22] developed a flight station assurance time planning model employing the Simple Temporal Network (STN) technique. Their approach incorporated time decoupling techniques, shortest path matrix simplification, and the STN task model distance graph solving method. This strategy represents an enhancement of the flight station assurance time collaborative planning, tailored for the airport environment, and takes into account resource factors comprehensively. Following the requirements of Collaborative Decision Making (CDM), Liu et al. [23] incorporated demands from air traffic control, airline operations, and airport operations. They introduced a position exchange strategy to systematically arrange inbound flights and, considering air traffic density factors, constructed a mathematical model for the collaborative sorting of incoming flights. The mainstream methods for coordinating airport surface resources are the two-phase model and bi-level model. In efforts to improve aircraft de-icing assurance efficiency, Su et al. [24] developed a two-phase model to coordinate the Aircraft Surface Operation (ASO) and De-icing Support Resource Operation (DSRO). To address aircraft conflict issues, Cerulli et al. [25] introduced two types of bi-level planning formulas and resolved them using a cutting generation algorithm. In a bid to minimize conflicts and waiting times between aircraft, Jiang [26] established a bi-level spatiotemporal taxiing optimization model to schedule aircraft taxiing start times and paths. Moreover, Jiang [27] proposed an optimization model for the airport Ground Movement Problem (GMP) based on bi-level planning, specifically designed to tackle taxiing conflict issues on airport grounds, thereby enhancing operational safety and efficiency. Similar collaborative mechanisms have also yielded substantial research achievements in other fields [28,29,30,31]. Due to the mutual influence of the upper and lower levels in bi-level planning, this can serve as a solution approach to coordinate between airport de-icing resources.

To sum up, among the existing de-icing models, the low utilization of slow-movement de-icing can be attributed to its high risk and relatively lower cost-effectiveness. Gate de-icing is predominantly employed at smaller airports, where de-icing resources are dispersed, leading to lower efficiency and challenges in de-icing fluid recovery, potentially causing adverse environmental impacts. On the other hand, centralized de-icing is the preferred choice at medium to large airports due to its efficient resource utilization, making it the optimal approach for airport de-icing operations. However, in the case of centralized de-icing, limitations exist in the current coordinated resource scheduling approaches. In existing centralized de-icing schemes, the constraints between position allocation and unmanned de-icing vehicle dispatch are not well-defined. While some schemes attempt to establish connections through variables, they can only ensure that position allocation impacts unmanned de-icing vehicle scheduling, without guaranteeing a significant reverse influence from unmanned de-icing vehicle scheduling to gate allocation, and vice versa. In simpler terms, if the focus is primarily on unmanned de-icing vehicle scheduling, the impact on de-icing position allocation is minimal, and vice versa. This indicates that a crucial constraint condition enabling true synergy between both sides of scheduling has not yet been identified.

In this study, we formulated a Mixed Integer Bi-Level Planning Model. In the upper level of the model, de-icing positions are allocated, whereas in the lower level, autonomous unmanned de-icing vehicles are scheduled. Therefore, the contributions of this paper are as follows:

(1) To establish a connection between parking position allocation and unmanned vehicle scheduling, we devised a specialized Mixed Integer Bi-Level Planning Model tailored for de-icing resource allocation. In the upper level of the model, the objective is to minimize the total flight delay time. This is accomplished by computing the wait time of each flight at the de-icing positions for the autonomous unmanned de-icing vehicles utilizing the vehicle scheduling and position allocation solutions. Subsequently, this wait time is employed as a constraint to optimize and derive a new de-icing position allocation scheme.
(2) In the lower level, utilizing the parking position allocation determined in the upper level, tasks are assigned to each unmanned de-icing vehicle team. Once tasks for each team are precisely defined, the wait time of each flight at the de-icing position for the autonomous unmanned de-icing vehicles is determined. This time is employed as a constraint influencing the solution at the upper level, enabling the model to prioritize flight allocation while optimizing vehicle travel distances.
(3) To tackle the challenge of traditional optimization algorithms potentially leading to flights departing too early or experiencing excessive delays, rendering the optimization solution impractical for real-world applications, we incorporate a Variable Neighborhood Search (VNS) strategy into the traditional genetic algorithm. By constraining the neighborhood scope, we mitigate the risk of large-scale changes in the flight sequence.
(4) Considering constraints associated with time, de-icing fluid capacity, and the travel distance of autonomous unmanned de-icing vehicles, we crafted a mixed multi-strategy improved heuristic greedy optimization approach. This strategy aims to minimize the instances of unmanned de-icing vehicles returning to their base, consequently reducing flight wait times and travel distances.

The remainder of the paper is structured as follows: in Section 2, we detail the formulaic definitions of the Mixed Integer Bi-Level Planning Model. Section 3 introduces an improved genetic algorithm combined with a VNS strategy to solve the de-icing position allocation problem and designs a mixed multi-strategy improved heuristic greedy optimization strategy to compute the optimal unmanned de-icing vehicle scheduling scheme. In Section 4, using data from a hub international airport in Northwestern China, the effectiveness of the model and the superiority of the algorithm are validated. Section 5 concludes the paper, providing insights into opportunities and directions for future research ideas.

## 2. Problem Description and Formulation

### 2.1. Problem Description and Model Assumptions

**Figure 1:**
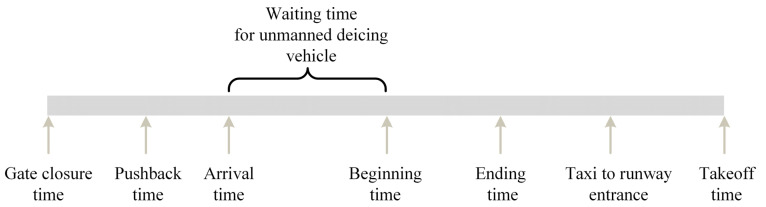
Aircraft de-icing timeline.

Figure 1 displays the complete timeline of a flight from the moment the cabin doors are closed to takeoff, including every instance of aircraft de-icing.

a. Description of the problem

**Figure 2:**
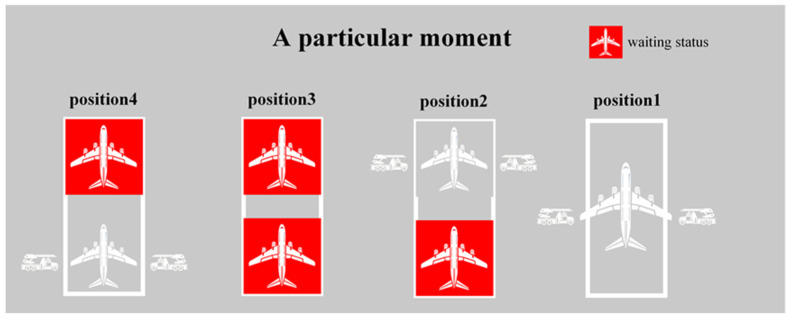
Aircraft waiting for unmanned de-icing vehicle.

**Figure 3:**
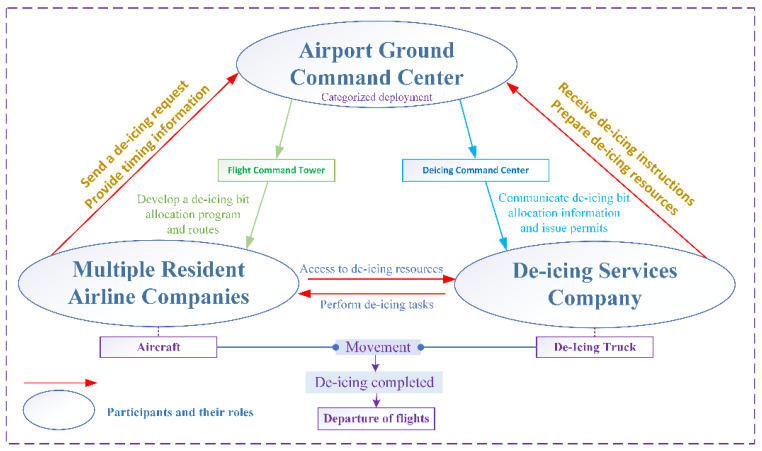
Roles and responsibilities of key de-icing participants.

This paper introduces a single-objective mixed-integer bi-level programming model. The upper-level model is responsible for allocating de-icing positions, while the lower-level model receives de-icing position allocation schemes from the upper-level model. It generates unmanned de-icing vehicle scheduling plans and calculates the waiting time for flights at de-icing positions based on both schemes. The obtained information is then fed back to the upper-level model in the form of constraints, facilitating the interaction between the upper- and lower-level models. Figure 2 illustrates a schematic representation of an Aircraft positioned at a de-icing position, awaiting the arrival of unmanned de-icing vehicles at a specific moment. Figure 3 depicts the relationships among the various stakeholders involved in the de-icing process and outlines their respective responsibilities.

b. Modeling assumptions

The following assumptions are proposed to simplify the model and reduce the complexity of problem handling:

(1) Aircraft with de-icing requirements adhere to the command center’s instructions, seamlessly navigating the prescribed path. They bypass delays in the flight segment caused by operational and assignment errors, minimizing the impact of human factors.
(2) Aircraft types are categorized into S, M, and L based on wingspan, fuselage length and height of the aircraft. The number of unmanned de-icing vehicles corresponding to different aircraft types is fixed at two.
(3) There are a total of *N* de-icing positions in the de-icing area, all of uniform type (i.e., the shape and area of the de-icing positions are the same). A single de-icing position can simultaneously carry out de-icing tasks for two S-type or M-type aircraft and one L-type aircraft.
(4) Aircraft, whether waiting for or completing de-icing, taxi to the runway immediately, without consideration of delays caused by queuing congestion or conflicts between aircraft and unmanned de-icing vehicles.
(5) Aircraft and unmanned de-icing vehicle speeds in the de-icing area are similar, without considering the variation speed of the aircraft on the runway.
(6) The total number of flights competing for de-icing positions (to be de-iced) is known and constant.
(7) Once the de-icing task is launched, the unmanned de-icing vehicle operation will not be interrupted.
(8) The volume of de-icing fluid consumed for a single flight varies from one aircraft type to another. It is a constant value that does not exceed the volume of the unmanned de-icing vehicle.
(9) Disregarding the reaction time of the de-icing fluid and the influence of meteorological factors, the de-icing fluid takes effect immediately upon being sprayed.
(10) The unmanned de-icing vehicles in operation will eventually return to the garage after achieving all de-icing tasks.

c. Description of symbols

Notations used in this model are presented in Table 1.

The list of variables utilized in this study is presented in Table 2

### 2.2. Bi-Level Modeling

This paper aligns with the comprehensive goals of airports seeking overall operational efficiency, airlines aiming at cost savings through reduced delays and local economic benefits, and ground service companies striving to minimize the operational costs of their unmanned de-icing vehicles. To address the diverse needs of these decision makers, this paper adopts a two-tiered, single-objective planning model.

#### 2.2.1. Upper-Level Modeling—Aircraft Scheduling Problem with Finite De-Icing Position Constraints

The upper layer model considers the minimization of the de-icing delay time as the objective function and realizes the rational scheduling of the aircraft to prevent this, i.e., to obtain a better result, by changing the allocation scheme of de-icing positions for the flight plan and constraining a limited number of de-icing positions and flight times. Therefore, it can be expressed as follows:

(1) Objective function:

(1) $$\min\sum_{i=1}^{N}\Delta T_{l_{i}}\;i\in N$$

where Δ*T_l_i__* denotes the length of delay time of flight *I*, which is expressed as follows:

(2) $$\Delta T_{l_{i}}=\left\{\begin{array}{cc} T_{reality\;i}-T_{plan\;i}, & \omega_{ik}=1\\ 0 & \omega_{ik}=0 \end{array}\right.\forall i\in N$$

(2) Constraints:

(3) $$F_{i(m)}^{t}\sigma_{i}^{M}\sigma_{i}^{L}=0\;\;\;F_{i(m)}^{t}\in \left\{0,1\right\}\;\forall i\in N,m\in M_{d}$$

(4) $$\left\{\begin{array}{c} 0\leq \sum_{t=1}^{T}\sum_{i=1}^{N}F_{i(m)}^{t}\sigma_{i}^{S}\leq 2\\ 0\leq \sum_{t=1}^{T}\sum_{i=1}^{N}F_{i(m)}^{t}\sigma_{i}^{M}\leq 2\\ 0\leq \sum_{t=1}^{T}\sum_{i=1}^{N}F_{i(m)}^{t}\sigma_{i}^{L}\leq 1 \end{array}\right.\;\;\;\sigma_{i}^{S},\sigma_{i}^{M},\sigma_{i}^{L}\in \{0,1\}\;\forall i\in N,m\in M_{d}$$

(5) $$\sum_{i=1}^{N}F_{i(m)}^{t}\leq 1\;\;\;\forall i\in N,\forall t\in T$$

(6) $$T_{early\;i}\leq T_{enter\;i}\;\;\forall i\in N$$

(7) $$s.t.\;T_{early\;i}=T_{\mathrm{plan}\;i}-\Delta T_{d_{i}}^{k}-\Delta T-\Delta T^{\prime }=T_{\mathrm{plan}\;i}-\Delta T_{hold}-\Delta T$$

(8) $$\Delta T_{d_{i}}^{k}=\Delta T_{d_{i}}^{S}\cdot \sigma_{i}^{S}+\Delta T_{d_{i}}^{M}\cdot \sigma_{i}^{M}+\Delta T_{d_{i}}^{L}\cdot \sigma_{i}^{L}$$

(9) $$T_{reality\;i}=T_{enter\;i}+\Delta T_{occupy\;i}+\Delta T^{\prime }$$

(10) $$\Delta T_{l_{i}}\leq \varepsilon_{i}$$

Constraints (3) and (4) dictate that aircraft types must be matched in the same de-icing position and should adhere to the de-icing pad’s accommodation conditions for the specific aircraft type. As for constraint (5), it stipulates that flights can only undergo de-icing operations in one de-icing position at any given time. Moreover, constraints (7) to (9) outline the relationships among the earliest feasible de-icing pad entry time, the de-icing duration for Type K flights, and the actual departure time of flights, encompassing various time variables. These equations lay the groundwork for calculating the time constraints in the upper-level model, denoted as constraint (6). Finally, constraint (10) signifies that the length of delay of a single flight cannot exceed the specified threshold.

#### 2.2.2. Lower Level Modeling—FSM-MDVRP for Unmanned De-Icing Vehicles

The lower-level model considers the adjustment of the path for unmanned de-icing vehicles based on changes in flight time. This section addresses the fleet size and mixed multi-depot vehicle routing problem for unmanned de-icing vehicles under the centralized de-icing mode at the airport. It accomplishes this by constructing a sophisticated multi-constraint MIP model with the objective of minimizing the travel distance of unmanned de-icing vehicles. Therefore, this system can be expressed as follows:

(1) Objective function:

(11) $$\min\;D=\min(D_{1}+D_{2})$$

(12) $$s.t.\;D_{1}=\sum_{v=1}^{L}\sum_{i=1}^{M_{d}}\sum_{j=1}^{M_{d}}H_{ij,v}D_{ij}\;\;\;H_{ij,v}\in \left\{0,1\right\}$$

(13) $$D_{2}=\sum_{v=1}^{L}\sum_{u=1}^{Q}\sum_{i=1}^{M_{d}}P_{ui,v}D_{ui}+\sum_{v=1}^{L}\sum_{j=1}^{M_{d}}\sum_{g=1}^{Q}P_{jg,v}D_{jg}\;\;\;P_{ui,v},P_{jg,v}\in \left\{0,1\right\}$$

(2) Constraints:

(14) $$\sum_{v=1}^{L}A_{iv}^{k}\cdot \sigma_{i}^{S}=Num\;S\;\;\;A_{iv}^{k},\sigma_{i}^{S}\in \{0,1\}$$

(15) $$\sum_{v=1}^{L}A_{iv}^{k}\cdot \sigma_{i}^{M}=Num\;M\;\;\;A_{iv}^{k},\sigma_{i}^{M}\in \{0,1\}$$

(16) $$\sum_{v=1}^{L}A_{iv}^{k}\cdot \sigma_{i}^{L}=Num\;L\;\;\;A_{iv}^{k},\sigma_{i}^{L}\in \{0,1\}$$

(17) $$\sum_{i=1}^{N}P_{ui,v}=1$$

(18) $$\sum_{j=1}^{N}P_{jg,v}=1$$

(19) $$\sum_{s}^{N}P_{is,v}=\sum_{s}^{N}P_{si,v}=A_{iv}^{k}\;\;\;\forall i\in N,\forall v\in L$$

In more detail, constraint (12) expresses the total distance traveled by the de-icing vehicles during task execution as the sum of the distances traveled by all unmanned de-icing vehicles while performing their respective tasks. In addition, constraint (13) represents the total distance traveled by the unmanned de-icing vehicles during depot departure and return as the sum of the distances traveled during their departures from and returns to the depot. Constraints (14)–(16) indicate that the number of de-icing vehicles matches the flight type. As for constraints (17) and (18), they denote that the unmanned de-icing vehicles put into service depart from the garage and eventually return to the garage. Finally, constraint (19) denotes that the flow of vehicles in and out of the de-icing position is conserved.

(20) $$T_{leave\;iv}-T_{begin\;i}\leq T_{hold\;i}\;\;\;\forall i\in N,\forall v\in L$$

(21) $$T_{arrive\;iv}\leq T_{begin\;i}\;\;\;\forall i\in N,\forall v\in L$$

(22) $$T_{end_{i}}=T_{gl_{i}}+\Delta T_{d_{i}}^{k}+\Delta T_{re_{i}}$$

(23) $$\Delta T_{re_{i}}=\left\{\begin{array}{cc} 2d_{ju}/v+\Delta T_{0} & \mathrm{The}\mathrm{unmanned}\mathrm{de-icing}\mathrm{vehicle}\mathrm{replenishes}\mathrm{the}\mathrm{de-icing}\mathrm{fluid}\\ 0 & otherwise \end{array}\right.$$

Constraints (20)–(22) represent the time constraints on the arrival and service of unmanned de-icing vehicles, where the arrival and departure times of the unmanned vehicles performing de-icing tasks are limited by flights.

When $\Delta T_{re_{i}}\neq 0$, the unmanned de-icing vehicle is loaded according to a volumetric relationship, and it is expressed as follows:

(24) $$\begin{array}{l} Q_{0}(Num\;S\cdot \sigma_{i}^{S}+Num\;M\cdot \sigma_{i}^{M}+Num\;L\cdot \sigma_{i}^{L})\\ =\sum_{i=1}^{N}\left(Q_{S}\cdot \sigma_{i}^{S}+Q_{M}\cdot \sigma_{i}^{M}+Q_{L}\cdot \sigma_{i}^{L}\right)+\sum_{v=1}^{L}Q_{iv}\cdot A_{iv}^{k} \end{array}$$

Then the conditions of $\Delta T_{re_{i}}\neq 0$ are as follows:

(25) $$\sum_{v=1}^{L}Q_{iv}\cdot A_{iv}^{k}<Q_{S}\cdot \sigma_{i}^{S}+Q_{M}\cdot \sigma_{i}^{M}+Q_{L}\cdot \sigma_{i}^{L}$$

(26) $$\sum_{v=1}^{L}Q_{iv}\cdot A_{iv}^{k}\cdot \sigma_{i}^{S}\leq Q_{0}$$

(27) $$\sum_{v=1}^{L}Q_{iv}\cdot A_{iv}^{k}\cdot \sigma_{i}^{M}\leq Q_{0}$$

(28) $$\sum_{v=1}^{L}Q_{iv}\cdot A_{iv}^{k}\cdot \sigma_{i}^{L}\leq Q_{0}$$

(29) $$\sum_{i=1}^{N_{p}}\sum_{v=1}^{L}A_{iv}^{k}\leq W$$

(30) $$\sum_{j=1}^{N}\sum_{v=1}^{L}P_{ju,v}\leq M_{\mathrm{limited}}$$

Constraints (26) to (28) show that the total volume of de-icing fluid loaded by the unmanned de-icing vehicle fleet is not less than the volume required for Type K flights. Moreover, constraint (29) indicates that the number of active unmanned de-icing vehicles cannot exceed the total number of available unmanned de-icing vehicles. In addition, constraint (30) specifies that the number of unmanned de-icing vehicles returning to the depot does not exceed the depot’s capacity.

## 3. Solving Algorithm

**Figure 4:**
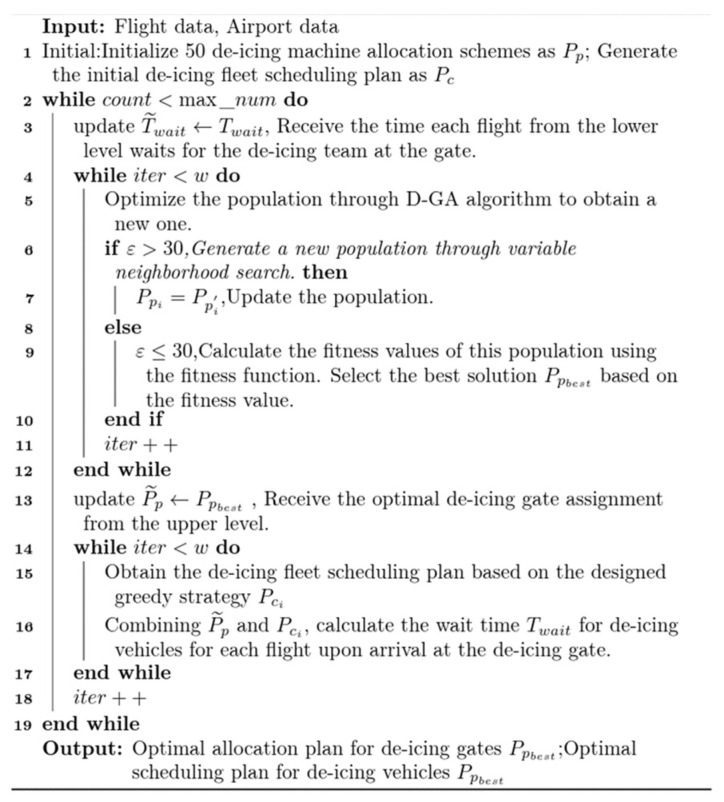
Main program pseudocode.

The main program steps are presented as shown in Figure 4. Initially, 50 de-icing position allocation schemes and de-icing vehicle scheduling schemes, based on the departure order, are initialized. At the higher level, the Mixed Variable Neighborhood Search Genetic Algorithm (MVNS-GA) is applied to obtain the de-icing position allocation scheme. This latter is then relayed to the lower level. Considering the scheme from the higher level as input, the lower level employs the Multi-Strategy Enhanced Heuristic Greedy Algorithm (MSEH-Greedy Algorithm) to determine the unmanned de-icing vehicle scheduling scheme. Applying this scheme in conjunction with the position allocation obtained from the upper level, the wait time for each flight for a de-icing vehicle is computed. This duration serves as a constraint and is sent back to the higher level. Therefore, the upper and lower models continuously exchange optimal solutions for single-level models and solve them until the termination conditions are met. The pseudocode of this algorithm is displayed in Figure 4, as follows:

(1) Upper level

**Figure 5:**
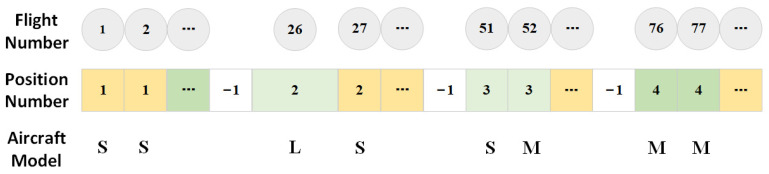
Example of the encoding method.

Encoding Method: the flight allocation problem within the model is encoded, converting problem space parameters into a format detectable by the computer. Based on the flight arrangement order, each flight is directed to one of the de-icing positions 1, 2, 3, or 4. By traversing all flights waiting for de-icing, a population is constituted, with all the flights arranged in sequence corresponding to their de-icing position numbers, as displayed in Figure 5.

Each position can simultaneously de-ice two S/M-type aircraft; however, it can only accommodate one L-type aircraft for de-icing. In the population depicted in Figure 5, flights 1 and 2, which are of M and S types, respectively, can be de-iced concurrently at position 1. Therefore, Flight 26, an L-type aircraft, occupies the entirety of position 2 for de-icing. The insertion of three “−1” entries splits the population into four sections, assigning each section’s de-icing tasks to one of the four positions mentioned previously.

**Figure 6:**
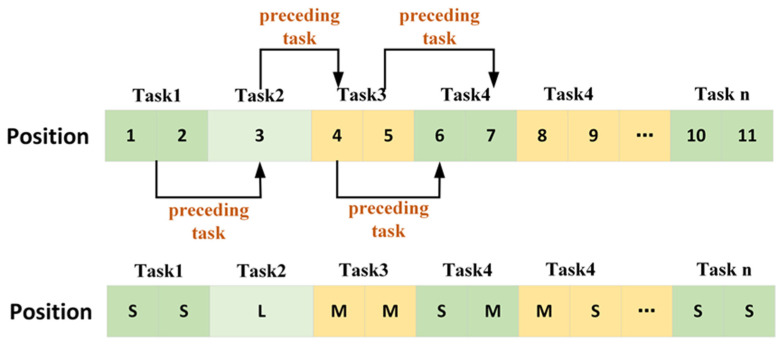
Task sequence diagram.

Furthermore, for each de-icing position, when an L-type aircraft is not involved, it can be regarded as two independent sections. Once the preceding task in each section is achieved, subsequent tasks enter the position. However, if there is an L-type aircraft, it can only enter the position after all flights in both preceding sections have accomplished de-icing and departed. Moreover, once the L-type aircraft has completed its de-icing, the next two aircraft can either enter the position simultaneously or one can enter followed by the second. To illustrate this act using a diagram (refer to Figure 6), Flight 3 is an L-type aircraft. Moreover, Flights 1 and 2 precede Flight 3; thus, Flight 3 can only enter the position once both Flights 1 and 2 are removed. Furthermore, Flight 3 precedes both Flights 4 and 5. Therefore, after Flight 3 finishes de-icing, both Flights 4 and 5 can either simultaneously or individually move to their new positions, where Flight 4 precedes Flight 6. After Flight 4’s de-icing is completed, Flight 6 can enter the section where Flight 4 was. Similarly, Flight 5 precedes Flight 7, and the pattern continues using this logic.

**Figure 7:**
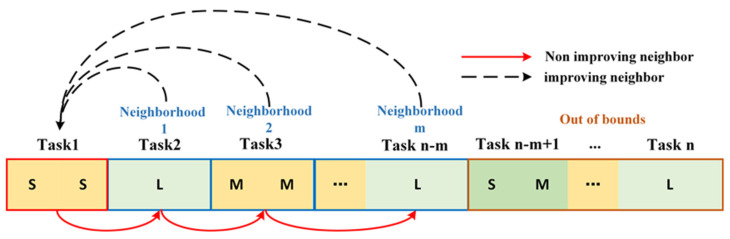
Constrained-variable neighborhood search strategy.

The upper level incorporates a variable neighborhood search strategy: to prevent a significant discrepancy between the actual and the planned departure times after flight sequence optimization, a VNS strategy is introduced during the population internal transformation process. Its transformation domain is also limited in scope. The schematic diagram is represented as shown in Figure 7.

When the search within the current neighborhood fails to yield a solution superior to the existing one, it progresses to the next neighborhood, as denoted by the red solid line in the diagram of Figure 7. However, upon discovering a superior solution within the current neighborhood, the search cycles back to the first neighborhood to commence anew, as illustrated by the black dashed line. To maintain a balance where flights neither depart too early nor experience excessive delays, the VNS is confined to a specific scope. Throughout the search process, transformations are only allowed within this predefined domain.

(2) Lower Level

**Figure 8:**
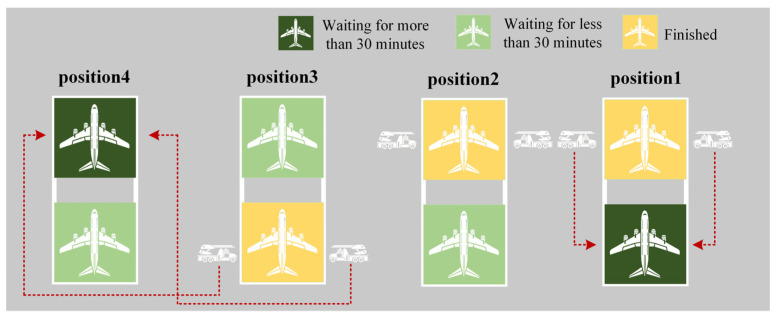
Time Strategy 1.

Utilizing a Mixed Multi-Strategy Enhanced Heuristic Greedy Optimization Strategy to determine the optimal de-icing vehicle scheduling plan: The essence of the greedy algorithm lies in selecting an appropriate greedy strategy. Given that the distance covered by a de-icing vehicle, when shuttling between positions, is significantly shorter than its return to the depot, the primary objective at the lower level is to maximize the usage of the de-icing fluid carried by each vehicle and minimize the number of returns to the depot while ensuring flight time priorities. Concurrently, the strategy should also aim at reducing the number of times a de-icing vehicle is rescheduled between positions to shorten its travel distance. Bearing this in mind, the following greedy strategies are expressed to determine the de-icing vehicle scheduling plan:

1. Time Strategy:

Upon completing the preceding tasks, the de-icing vehicle traverses through all flights waiting at the four de-icing positions. If a flight has been waiting for over 30 min (in accordance with the algorithm constraint limiting an individual flight’s delay to 30 min), the nearest available vehicle team prioritizes that specific flight. The schematic representation is as follows: the closest free vehicle team heads to the nearest flight that has been waiting for more than 30 min. A schematic illustration of the Time Strategy is presented in Figure 8.

**Figure 9:**
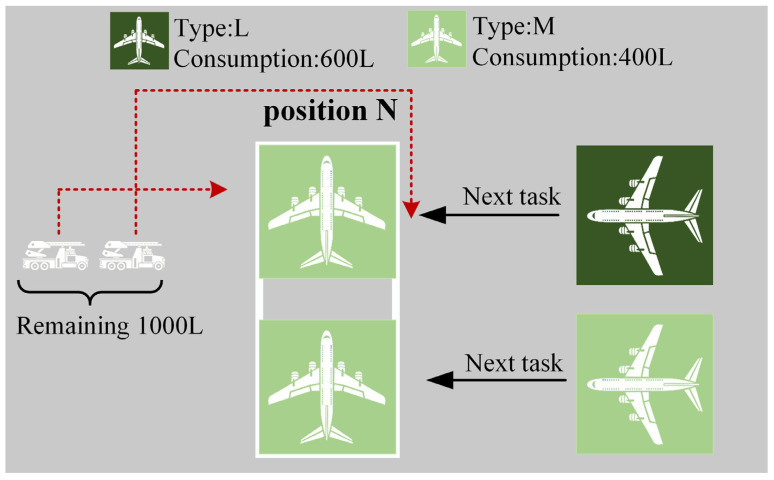
De-Icing Fluid Strategy 1.

**Figure 10:**
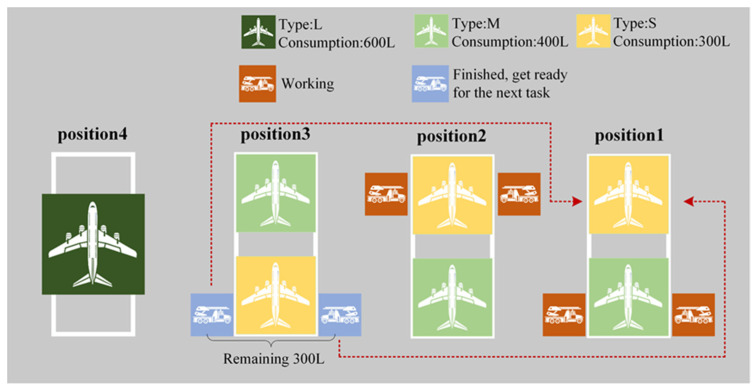
De-Icing Fluid Strategy 2.

**Figure 11:**
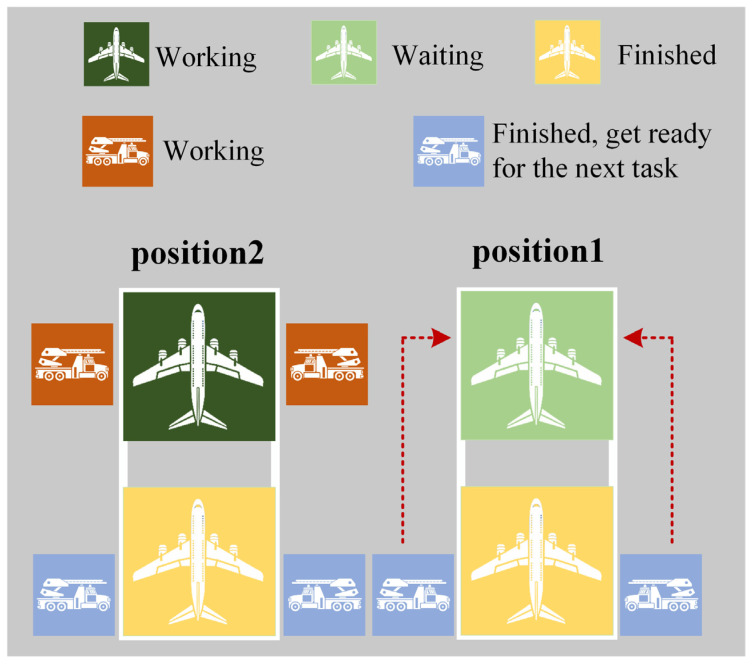
Distance Strategy 1.

**Figure 12:**
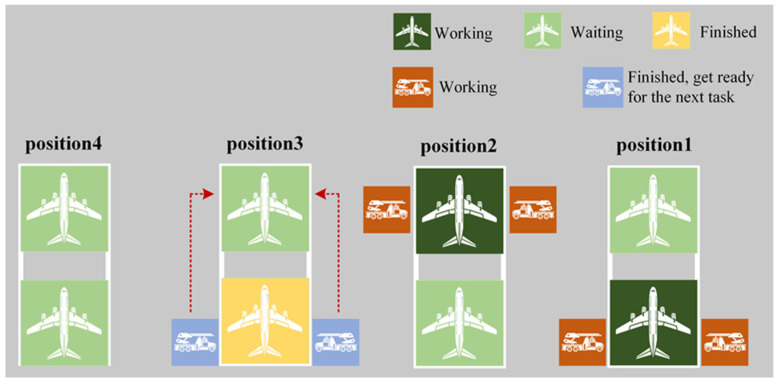
Distance Strategy 2.

2. De-Icing Fluid Strategy:
(1) Adequate De-Icing Fluid: following the completion of the de-icing vehicle’s preceding tasks, and when faced with two or more available tasks for selection, the vehicle prioritizes the de-icing task at the target position. More precisely, it selects the task (and its subsequent task) where the total de-icing fluid consumption is the closest to the remaining de-icing fluid quantity in the vehicle. A schematic illustration of the De-Icing Fluid Strategy 1 is presented in Figure 9.

(2) Insufficient De-Icing Fluid: Upon completing its preceding tasks, if the remaining de-icing fluid is insufficient to complete the tasks for the aircraft waiting at the same position, the de-icing vehicle searches for any other flight currently waiting at the de-icing positions that can be addressed with the available fluid. Upon finding such a flight, the vehicle de-ices that flight and, consequently, returns to the depot. A schematic illustration of the De-Icing Fluid Strategy 2 is presented in Figure 10.

3. Distance Strategy:
(1) When considering a specific flight awaiting de-icing, the closest available vehicle team, in terms of distance to the target task, is chosen to proceed to that position for de-icing. A schematic illustration of the Distance Strategy 1 is presented in Figure 11.

(2) In the case of a specific de-icing vehicle team, if there are multiple subsequent de-icing tasks meeting the aforementioned criteria, the task closest in distance is chosen for execution. A schematic illustration of the Distance Strategy 2 is presented in Figure 12.

**Figure 13:**
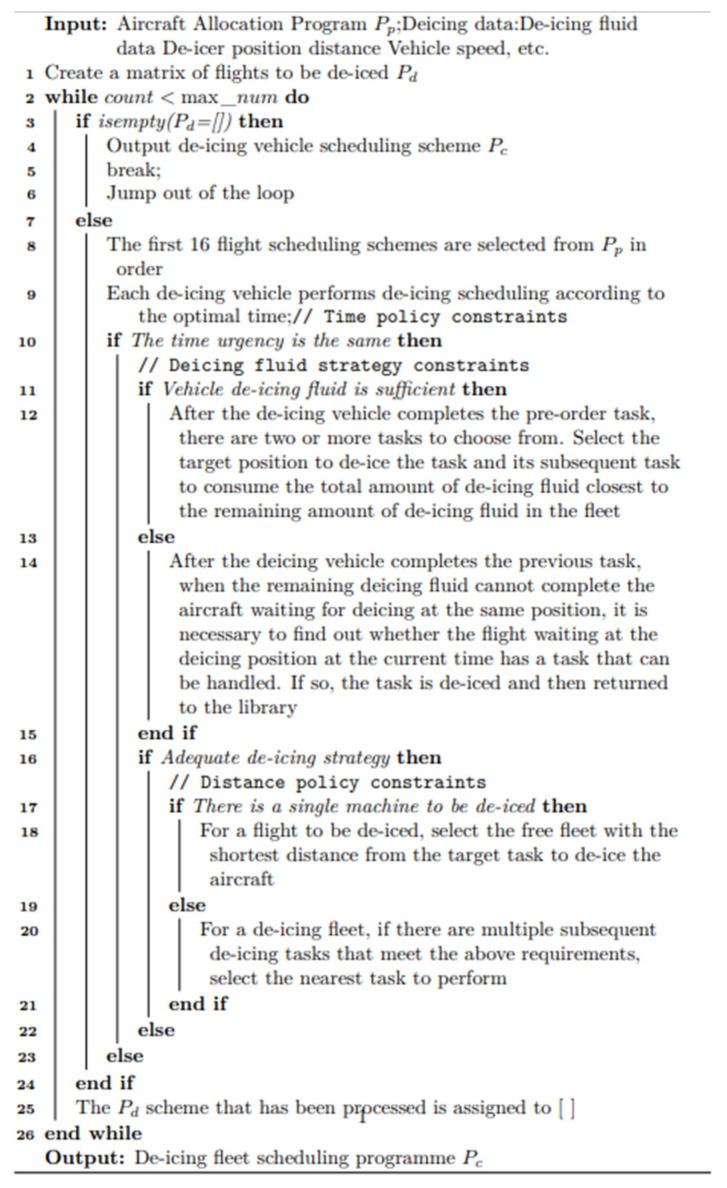
Greedy strategy pseudocode.

The pseudo-code representation of the above greedy strategies is displayed in Figure 13, as follows:

## 4. Experiments and Analysis

This study conducted tests on the mixed-integer programming two-tier model and algorithm using data from 100 flights scheduled between 06:40 and 12:40 on 31 January 2023 at a specific international airport in the northwest.

In the Section 4, a de-icing scheme based on the departure sequence is firstly computed. Then, the total delay time for all flights in this scheme is calculated along with the corresponding unmanned de-icing vehicle dispatch plan. This scheme will serve as a benchmark for subsequent model and algorithm validation. Subsequently, our improved algorithm is applied to solve the de-icing position allocation as well as the unmanned de-icing vehicle dispatch plan. When solving with the MVNS-GA algorithm, several similar frontier algorithms are employed to compare and validate the effectiveness of the proposed algorithm. Moreover, the MSEH-Greedy Algorithm is applied to solve the unmanned de-icing vehicle dispatch plan obtained using different algorithms in the upper-level model. It is important to note that the average and maximum waiting times for flights at de-icing positions are calculated and saved for further analysis.

Moreover, the algorithm implementation is carried out using MATLAB R2021a on a Windows 10 operating system having an NVIDIA GeForce GTX 1050 Ti graphics card. It is worth noting that the defined de-icing resources consist of a total of four de-icing positions, each capable of accommodating two S- or M-type aircraft for de-icing, and only one L-type aircraft at a time. Furthermore, there are three unmanned de-icing vehicle fleets, each equipped with two de-icing vehicles for aircraft de-icing. During operations, the de-icing fluid consumption is distributed evenly over each fleet. The process involves fleet assembly at the de-icing area followed by individual aircraft de-icing at respective positions. These settings do not influence the validation of the models or the algorithms proposed in this study.

Table 3 presents the de-icing tasks for each position using the flight departure order, i.e., the de-icing schedule is based on the planned departure sequence of the day. The sequence number describes the original departure order; for instance, (1, 5) indicates that aircraft 1 and 5, belonging to the S\M models, are being de-iced at position 1 simultaneously at a given moment. Table 3 displaying only a subset of tasks, uses ‘...’ to indicate tasks that are omitted and not shown.

Under these study conditions, during the experimental timeframe, the considered 100 flights experienced a cumulative delay of 3128 min.

Table 4 presents the scheduling plan for the unmanned de-icing vehicles based on the given de-icing position allocation scheme. For example, ‘De-icing Position 1 → De-icing Position 1’ represents the movement of an unmanned de-icing vehicle within the same position. However, ‘De-icing Position 1 → De-icing Position 4’ signifies the unmanned de-icing vehicle transitioning from position 1 to position 4. According to this initial plan, the unmanned de-icing vehicles returned to the garage a total of 15 times, traveling a joint distance of 34,500 m. Table 4 displaying only a subset of tasks, uses ‘...’ to indicate tasks that are omitted and not shown.

### Experimental Results

In the simulation experiment comparison, several typical cutting-edge optimization algorithms were selected, such as Genetic, Differential Evolution, Particle Swarm Optimization, Grey Wolf Optimizer, and Raven Search algorithms for horizontal comparison. This was performed to validate the global search capability, convergence, and robustness of the Hybrid Variable Neighborhood Search Genetic Algorithm.

**Figure 14:**
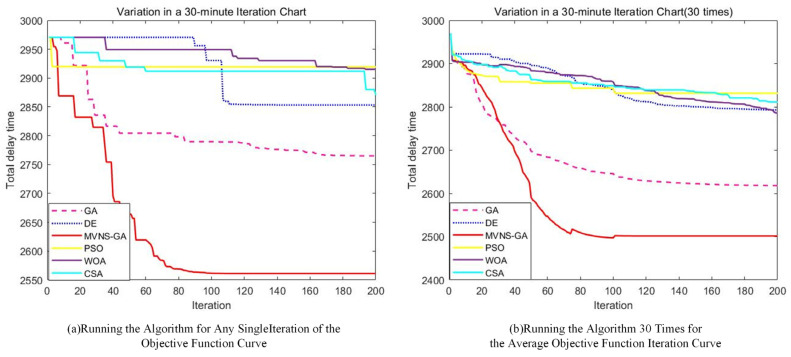
Upper-level iteration curve chart 1.

In the algorithmic solution regarding the total delay time of the upper model, the delay times obtained by the different optimization algorithms are provided in Table 5. Compared to de-icing in the order of departure, the total delay time of MVNS-GA decreased by 567 min, yielding a reduction of 18.12%. In contrast, the DE, PSO, WOA, CSA, and GA algorithms reduced the total delay times by 275, 209, 213, 258, and 363 min, respectively, representing reductions of 8.79%, 6.68%, 6.81%, 8.25%, and 11.64%. Moreover, compared to the reference algorithms, the improved algorithm reduced the total delay times by 292, 358, 354, 309, and 204 min, respectively. Notably, in comparison with the traditional GA algorithm, there was a reduction of 204 min. This result reflects the effectiveness of the enhanced algorithm when solving the mixed-integer programming two-tier model. During the solution process, the iterative evolutionary processes between the two levels of the model for both the improved algorithm and the comparative algorithms are displayed in Figure 14. As the iteration progressed, the optimal total flight delay time exhibited a fluctuating downward trend between the 0th to 104th iterations. After 107 iterations, the decrease became gradual and slowly stabilized, approaching the optimal solution.

Table 6 presents the optimal de-icing position scheduling solution obtained using the Hybrid Variable Neighborhood Search Genetic Algorithm.

**Figure 15:**
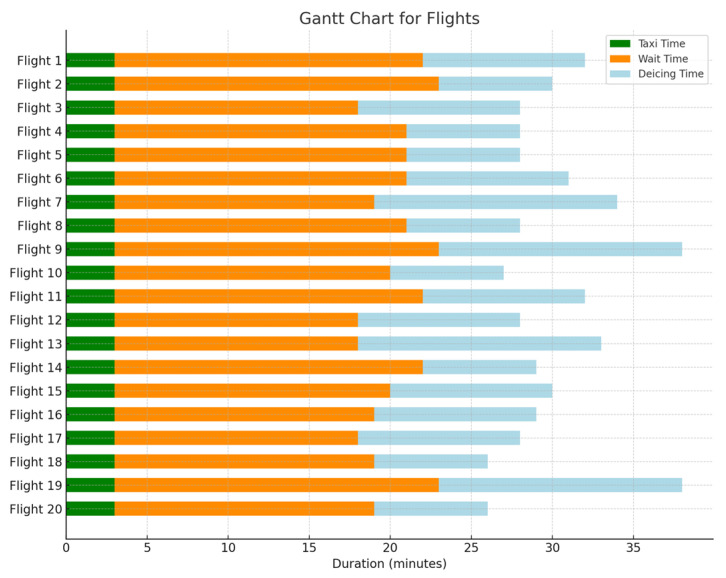
Initial proposal Gantt chart.

**Figure 16:**
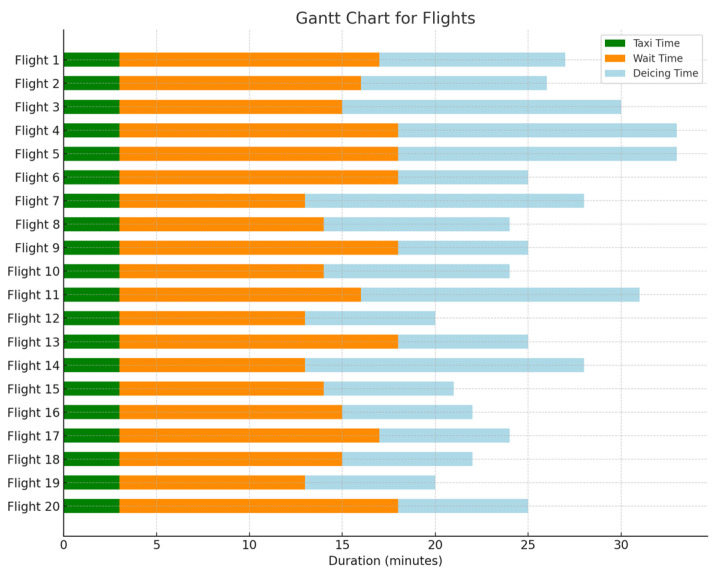
Final proposal Gantt chart.

Figure 15 and Figure 16 illustrate the Gantt chart for the 20 flights with the longest delay durations in both the initial and optimized scenarios. In this chart, the green color represents the taxiing time, the orange color indicates the waiting time, and the blue color denotes the de-icing time. This reduction in the waiting time for flights due to unmanned de-icing vehicles is noticeable. The average waiting time for the 20 flights with the longest delay durations in the initial scenario is 18.5 min per aircraft, whereas in the optimized scenario, it is reduced to an average of 12.8 min, resulting in an average reduction of 5.7 min. Thus, it is evident that the model and algorithm established in this study effectively decrease the waiting time for flights at de-icing positions.

**Figure 17:**
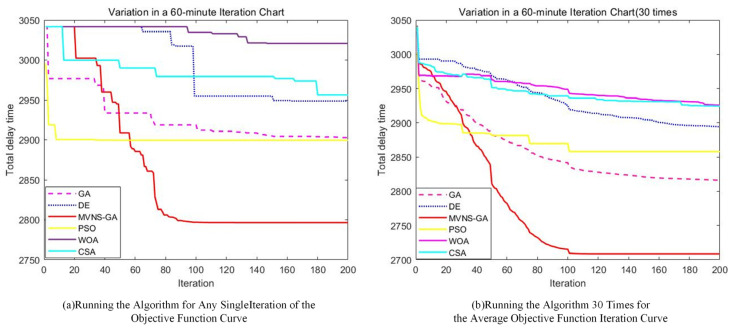
Upper-level iteration curve chart 2.

To investigate the potential impact of expanding the time variation range for a single flight on the total delay time for all flights, we modified the model to allow a flight’s departure to be advanced or delayed by up to 60 min, doubling the previous limit of 30 min. Employing the same algorithm for the solution, the iterative results are depicted in Figure 17.

Comparing the above results(The specific data is presented in Table 7.) with the solution that limits the adjustment to 30 min, it is clear that the optimal solutions of the DE, WOA, CSA, GA, and MVNS-GA algorithms, when the flight adjustment interval is set to 60 min, are worse than when setting it to 30 min, resulting in increased delay times. Only the PSO algorithm’s optimal solution yielded in better results than those obtained with a 30 min adjustment interval. This is because when flights are allowed a larger adjustment range, it can lead to excessively long delays for some flights, thereby increasing the total delay time. Therefore, it is advisable to set the flight adjustment range to 30 min.

Whenever the upper level attains an optimal de-icing plan and conveys it to the lower level, a scheduling plan for the unmanned de-icing vehicle is derived through a well-designed greedy strategy. For the upper model solved by different algorithms, distinct lower-level vehicle scheduling plans are derived. After the upper model is solved using MVNS-GA and passed to the lower level, the shortest distance traveled by the unmanned de-icing vehicles is 30,783 m. As for the GA, DE, PSO, WOA, and CSA algorithms, after solving the upper model and passing it to the lower level, the distances traveled by the unmanned de-icing vehicles are 31,972 m, 34,138 m, 34,273 m, 33,634 m, and 33,837 m, respectively. In the final unmanned de-icing vehicle scheduling plan, the vehicle’s total travel distance was reduced by 3717 m, whereas the number of return trips to the garage decreased by one. The final scheduling plan for unmanned de-icing vehicles is presented in Table 8

**Figure 18:**
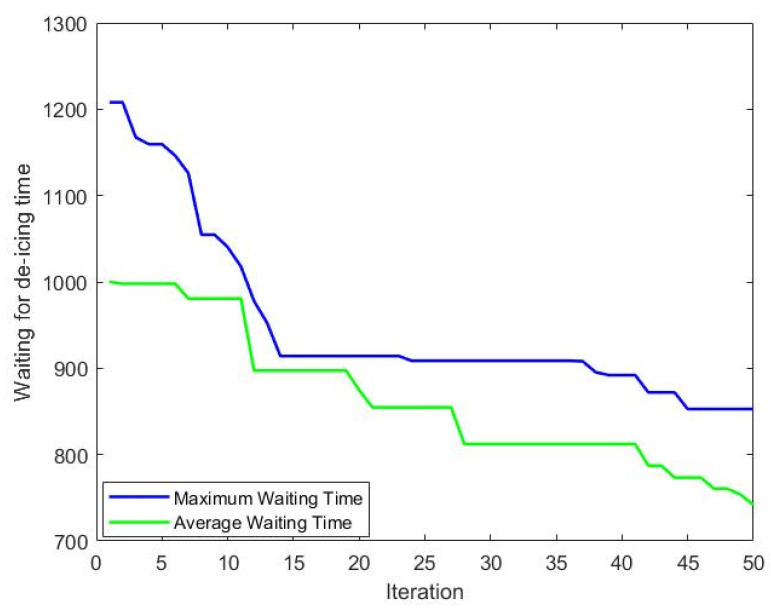
Wait time variation with iteration graph.

Whenever the lower level receives an allocation plan from the upper level and determines a scheduling plan for the unmanned de-icing vehicles, it can calculate the distance of each flight in the received plan required while waiting for an unmanned de-icing vehicle. This wait time is a critical period influencing the actual take-off time of the aircraft. As the main function iterates, the time aircraft wait at de-icing positions for unmanned de-icing vehicles decreases. By the end of the iterations, the waiting time for de-icing after the arrival of each aircraft at the de-icing position decreased from approximately 1005 s to 732 s. Variations in the maximum and average waiting times with the number of iterations are depicted in Figure 18.

The simulation experiments mentioned above validated the effectiveness of the proposed mixed-integer dual-layer planning model in this paper, highlighting the superiority of the MVNS-GA algorithm. Notably, it reduced the waiting arrival time of flights for unmanned de-icing vehicles and utilized this time as a bridging variable between the upper and lower layers of the model. This ensures the optimal allocation of de-icing positions while also achieving the optimal operation plan for unmanned de-icing vehicles.

## 5. Conclusions

This paper tackles the problem of inefficient airport flight position allocation and suboptimal operation scheduling of unmanned de-icing vehicles. With the goals of minimizing total flight delay time and reducing the travel distance of unmanned de-icing vehicles, we took into account the actual flight constraints of a specific airport in the northwest. To address these challenges, we developed a mixed-integer dual-layer planning model for optimizing airport de-icing resources. In more detail, the upper layer of this model provides a distribution plan for de-icing positions, whereas the lower layer ensures an optimal operation plan for unmanned de-icing vehicles. This combination facilitates the coordinated operations between the de-icing position scheduling and the vehicle operations. In our algorithm design, a genetic algorithm combined with a variable neighborhood search for solving the upper layer is introduced. By restricting the range of neighborhood changes, the total delay time under different single flight time constraints is calculated, ensuring a logical order of flight adjustments while reducing the total delay time. Moreover, the lower layer’s solution uses a greedy strategy that integrates time policies, de-icing fluid strategies, and path length strategies. This approach efficiently identifies scheduling solutions for unmanned de-icing vehicles, minimizing both the flight waiting times and the path lengths. Compared to de-icing based on the departure order, the proposed mixed-integer dual-layer planning model and two-stage solution algorithm effectively reduce flight delay times by 567 min and vehicle travel distances by 3717 m, achieving the objective of improving airport de-icing efficiency. This result fosters the potential of applying bio-inspired algorithm optimization to unmanned equipment in airport de-icing operations.

While this paper achieved some progress in optimizing airport de-icing resources, several issues remain unresolved for future research. In our simulation experiments, we simplified the queuing conditions before and after de-icing; however, we did not consider unforeseen circumstances. In real-world scenarios, unexpected events are crucial, such as emergency queue jumps by military or other aircrafts for de-icing. Therefore, in future studies, we will focus on how the queuing of flights at the start and end of de-icing affects departure times and consider emergency plans in unforeseen situations.

## Figures and Tables

**Table 1:** Notations and their description.

Set and Parameter Notations	
T	Set of flight time, $T=\left\{T_{p_{1}},T_{p_{2}},T_{p_{3}},\ldots ,T_{p_{Q}}\right\}$
N	Set of flights, total Q flights, $N=\left\{p_{1},p_{2},\ldots ,p_{f},\ldots ,p_{Q}\right\}$
$M_{d}$	Set of de-icing positions, total E de-icing positions,$M_{d}=\left\{r_{1},r_{2},\ldots ,r_{i},\ldots ,r_{E}\right\}$
L	Set of unmanned de-icing vehicles, total *W* unmanned de-icing vehicles, $L=\{v_{1},v_{2},\ldots ,v_{W}\}$
Q	Set of de-icing depots, total B depots, $Q=\{r_{1},r_{2},\ldots ,r_{u},\ldots ,r_{B}\}$
O	Set of flight types, k∈O={S,M,L}
$T_{early\;i}$	Earliest available time for flight i to taxi to the de-icing ramp
$T_{enter\;i}$	Moment when flight i moves onto the de-icing ramp
$T_{\mathrm{plan}\;i}$	Scheduled departure time of flight i
$T_{reality\;i}$	Actual time of departure of the flight i
$T_{leave\;iv}$	Moment when unmanned de-icing vehicle v leaves de-icing position in service of flight i
$T_{arrive\;iv}$	Moment of arrival of unmanned de-icing vehicle v at de-icing position in service of flight i
$\Delta T_{d_{i}}^{k}$	De-icing time for a single k flight
$\Delta T_{occupy\;i}$	Occupancy time for flight i
$\Delta T_{hold\;i}$	Holding time of de-icing fluid
ΔT	Time for flights to slide from the waiting area into the de-icing position, constant value
$\Delta T^{\prime }$	Time for flights to taxi from the de-icing position to the runway, constant value
$\Delta T_{l_{i}}$	Length of aircraft delay: when a flight is delayed, the value is $T_{reality\;i}-T_{plan\;i}$; otherwise, it is zero
$\Delta T_{0}$	Hours for de-icing fluid replenishment by one unmanned de-icing vehicle
$\Delta T_{re}$	Hours for fluid replenishment for a single unmanned de-icing vehicle
$\varepsilon_{i}$	Thresholds for the length of delay of flight i, i.e., maximum delays
Num S	Unmanned de-icing vehicle size for *S*-type aircraft
Num M	Unmanned de-icing vehicle size for *M*-type aircraft
Num L	Unmanned de-icing vehicle size for *L*-type aircraft
v	Average travel speed of unmanned de-icing vehicles
$d_{ju}$	Distance from de-icing position j to special garage u
D	Total distance traveled by unmanned de-icing vehicles
$D_{1}$	Distance traveled by unmanned de-icing vehicles on mission
$D_{2}$	Driving distances for unmanned de-icing vehicles out of and back to the depot
$Q_{0}$	Maximum volume of de-icing fluid that can be carried in an unmanned de-icing vehicle
$Q_{iv}$	Volume of de-icing fluid for unmanned de-icing vehicle v providing de-icing services for flight i
$Q_{k\in O}$	Volume of de-icing fluid consumed by a single de-icing sortie of a type k flight
$M_{\mathrm{limited}}$	Volume of a single de-icing garage

**Table 2:** List of variables used in this study.

Set and Parameter Notations	
$\omega_{i}^{k}$	$\omega_{i}^{k}\in \{0,1\}$, when $\omega_{i}^{k}=1$, $T_{reality\;i}-T_{delay\;i}>0$ indicates that the flight i is delayed; otherwise $\omega_{i}^{k}=0$, this moment $T_{reality\;i}-T_{delay\;i}\leq 0$
$F_{i(m)}^{t}$	$F_{i(m)}^{t}\in \left\{0,1\right\}$, when $F_{i(m)}^{t}=1$, flight i is de-icing at de-icing position n at time t; otherwise, it is equal to zero
$\sigma_{i}^{k}$	$\sigma_{i}^{k}\in \{0,1\}$, when $\sigma_{i}^{k}=1\;(k\in O)$, type of airplane is k; otherwise, zero
$A_{iv}^{k}$	$A_{iv}^{k}\in \{0,1\}$, when $A_{iv}^{k}=1$, indicates that unmanned de-icing vehicle *v* serves flight i of type k; otherwise, zero
$H_{ij,v}$	$H_{ij,v}\in \left\{0,1\right\}$ ∀i,j∈N, when $H_{ij,v}=1$, indicates that the unmanned de-icing vehicle v travels from de-icing position i to de-icing position j; otherwise, zero
$P_{ui,v}$	$P_{ui,v}\in \left\{0,1\right\}$ ∀u∈Q,i∈N, when $P_{ui,v}=1$, indicates that the unmanned de-icing vehicle v travels from garage u to de-icing position i; otherwise, zero
$P_{jg,v}$	$P_{jg,v}\in \left\{0,1\right\}$ ∀g∈Q,j∈N, when $P_{jg,v}=1$, indicates that the unmanned de-icing vehicle v travels from de-icing position j to garage g; otherwise, zero

**Table 3:** Initial position assignment plan.

Position	Task 1	Task 2	Task 3	…	Task n
1	(1, 5)	9	(15, 17)	…	(94, 98)
2	(2, 6)	(10, 13)	(18, 21)	…	(95, 99)
3	(3, 7)	(11, 14)	(19, 23)	…	(96, 100)
4	(4, 8)	(12, 16)	(20, 24)	…	(93, 97)

**Table 4:** Initial gate assignment plan.

Team	Tasks	Number of Returns to Garage
1	De-icing Position 1 → De-icing Position 1 → De-icing Position 4 → … → De-icing Position 4	5
2	De-icing Position 2 → De-icing Position 2 → De-icing Position 3 → … → De-icing Position 3	4
3	De-icing Position 3 → De-icing Position 3 → De-icing Position 2 → … → De-icing Position 4	5

**Table 5:** Comparative algorithm between the different methods.

Method	Total Delay Time (min)
De-icing in Departure Sequence	3128
Differential Evolution (DE)	2853
Particle Swarm Optimization (PSO)	2919
Whale Optimization Algorithm (WOA)	2915
Crow Search Algorithm (CSA)	2870
Genetic Algorithms (GA)	2765
MVNS-GA	2561

**Table 6:** Optimized allocation plan.

Position	Task 1	Task 2	Task 3	…	Task n
1	(1, 6)	(12, 13)	(15, 29)	…	(94, 95)
2	(2, 8)	9	(19, 27)	…	(97, 100)
3	(3, 6)	(11, 18)	(21, 23)	…	(98, 99)
4	(4, 10)	(14, 17)	(22, 26)	…	(93, 96)

**Table 7:** Comparison chart of optimization effects in different ranges.

Method	Total Delay Time (Min)	Compared to 30 Minutes (Min)
DE	2948	+95
PSO	2899	−20
WOA	3020	+105
CSA	2956	+86
GA	2902	+137
MVNS-GA	2796	+235

**Table 8:** Final scheduling plan for unmanned de-icing vehicles.

Team	Tasks	Number of Returns to Garage
1	De-icing Position 1 → De-icing Position 2 → De-icing Position 2 → … → De-icing Position 3	4
2	De-icing Position 1 → De-icing Position 2 → De-icing Position 3 → … → De-icing Position 1	4
3	De-icing Position 3 → De-icing Position1 → De-icing Position 4 → … → De-icing Position 2	5

## Data Availability

Data is contained within the article.
